# Evaluation of the quality of service delivery in private sector, primary care clinics in Kenya: A descriptive patient survey

**DOI:** 10.4102/safp.v62i1.5148

**Published:** 2020-10-22

**Authors:** Gulnaz Mohamoud, Robert Mash

**Affiliations:** 1 Department of Family Medicine, Aga Khan University Hospital, Nairobi, Kenya; 2 Division of Family Medicine and Primary Care, Stellenbosch University, Cape Town, South Africa

**Keywords:** consultation, General Practice Assessment Questionnaire (GPAQ), health care quality, Kenya, patient satisfaction, primary care, private sector, service delivery

## Abstract

**Background:**

The quality of service delivery in primary care (PC) is an important determinant of clinical outcomes. The patients’ perspective is one significant predictor of this quality. Little is known of the quality of such service delivery in the private sector in Kenya. The aim of the study was to evaluate the quality of service delivery in private sector, PC clinics in Nairobi, Kenya.

**Methods:**

The study employed a descriptive cross-sectional survey by using the General Practice Assessment Questionnaire in 378 randomly selected patients from 13 PC clinics. Data were analysed using the Statistical Package for Social Sciences.

**Results:**

Overall, 76% were below 45 years, 74% employed and 73% without chronic diseases. Majority (97%) were happy to see the general practitioner (GP) again, 99% were satisfied with their consultation and 83% likely to recommend the GP to others. Participants (97%) found in receptionist helpful and the majority were happy with the opening hours (73%) and waiting times (85%). Although 84% thought appointments were important, only 48% felt this was easy to make, and only 44% were able to access a particular GP on the same day. Overall satisfaction was higher in employed (98%) versus those unemployed (95%), studying (93%) or retired (94%) (*p* < 0.001).

**Conclusion:**

Patients reported a high quality of service delivery. Utilisation was skewed towards younger, employed adults, without chronic conditions, suggesting that PC was not fully comprehensive. Services were easily accessible, although with little expectation of relational continuity. Further studies should continue to evaluate the quality of service delivery from other perspectives and tools.

## Background

The World Health Organization (WHO) asserts that ‘access to timely, acceptable, affordable, and high quality health care is a fundamental right of every human being’.^1^ Health care systems have better health outcomes when built on primary health care (PHC), where prevention and promotion are in balance with curative interventions and ‘appropriate referral to higher levels of care’.^2,3,4^ World Health Organization subdivides the PHC approach into four main areas: universal health coverage (UHC), sound policies, governance and leadership and primary care (PC).^5^

Primary care is defined in terms of its ‘four functions which are, first contact access for every health need; long-term person-focussed care, comprehensive and coordinated care that is measurable and its quality assessed’.^6,7^ Therefore, there is a need to measure the quality of service delivery so that strategies can be put in place to further improve and strengthen PC.^6^ One way of evaluating the quality of PC is by obtaining feedback from the patients regarding the practice, their consultations and practitioners.^8^ Satisfaction of patients is a key predictor of the quality of service delivery.^8,9^ Hence, identifying the gaps in quality of PC service delivery will help to achieve the goals of PHC.^6^

In PC, communication skills are as critical as the generalists’ clinical competency for an effective and satisfactory consultation.^10^ Several studies have shown that communication is one of the most important factors contributing towards overall satisfaction.^11,12,13^ The degree to which patients’ expectations of their consultations are fulfilled has a strong bearing on their satisfaction and the perceived quality of service delivery.^14^ Consultations should enable patients to understand their health problems, adhere to their management plan and take control of their illness.^15,16,17^ Communication skills should support a broad and wholistic bio-psycho-social or person-centred approach to the consultation to deliver high-quality PC.^18^ Communication and consultation skills are also important for the trust and confidence that patients have in their PC provider.^7,19,20^

Easy access to care is another important factor that impacts on satisfaction separately from the consultation itself.^21^ High-quality service delivery in PC should also enable continuity of care over multiple illness episodes and coordinate care for the individual between different teams and levels of care.^7,20^ Primary care should also deliver a comprehensive package of care from conception to end-of-life care and across the burden of disease.^7^

The quality of service delivery can, therefore, be assessed by attention to the quality of the consultation and person-centredness, access to care, continuity of care, coordination of care and comprehensiveness.^7^ A systematic review in sub-Saharan Africa (SSA) listed ‘access and cost of care, doctor-patient relationship, and healthcare resources as main contributors to patient satisfaction’.^22^ Studies conducted within East Africa have linked satisfaction to communication, empathy, cleanliness, adequacy of medical supplies, technical equipment and staff attitudes.^23,24,25^ These studies show consistently high levels of satisfaction despite well-documented inadequacies, such as lack of essential resources, medication, equipment and shortages of personnel.^23^

The relationship between patient satisfaction and quality of care is complex because other factors such as expectations play an important role.^26^ Nevertheless, patient satisfaction remains a significant aspect of understanding the quality of care in service delivery because patients are ultimately the clients.

In addition to expectations, socio-demographic factors may also predict patient satisfaction, although results are not consistent.^24,27^ A study at a district hospital in the public sector of Kenya found that older married men were more satisfied, whereas a study from a family medicine clinic in a Nigerian teaching hospital found no such relationship.^13,24^

The health system in Kenya has three categories of service providers: public sector services (48%), not-for-profit private organisations (14%) that includes religious, mission hospitals and non-governmental organisations [NGOs] and private-for-profit providers (38%).^28^ Therefore, the private sector provides 52% of health services in Kenya and this proportion is growing.^28^ Understanding the quality of service delivery in the private sector is important.

A few studies in Africa have evaluated the quality of service delivery from the patient’s perspective and no studies were identified from the private sector in Kenyan PC.^22^ This study therefore will bridge the gap in our knowledge of PC in the African context and should help to identify ways of improving service delivery in this context. The aim of this study was to evaluate the quality of service delivery from the patients’ perspective in private sector, PC clinics in Nairobi, Kenya.

## Methods

### Study design

This was a descriptive cross-sectional survey, using the General Practice Assessment Questionnaire revalidated version 2 (GPAQ-R2).

### Setting

Nairobi, the capital city of Kenya is home to approximately 3.5 million people, which is almost 10% of the country’s population.^29^ A private tertiary care hospital was linked with 13 PC clinics in Nairobi County, which were run by general practitioners (GPs). These ambulatory PC facilities offered services in semi-urban, urban and peri-urban areas of Nairobi. Most of the clinics were operational throughout the week and were open at times suited to an employed population. They catered for all age groups and services included health promotion, disease prevention and curative treatment. The clinics also included registered nurses, pharmacy technicians, laboratory technicians, radiographers and receptionists. On an average, 35 patients were seen at these clinics per day, and most of them were covered by private medical insurance by virtue of their employment.

The tertiary hospital associated with these PC clinics also had a Department of Family Medicine, which was run by specialist family physicians. They offered out-patient family medicine services alongside the usual hospital specialists and sub-specialists and received referrals from the PC clinics. The PC clinics had easy access to refer patients to family medicine, the accident and emergency centre or other specialities at the tertiary hospital. There was no compulsory gatekeeping at the PC level, and patients could choose to access care via the PC clinics or the hospital.

### Study population and sample size calculation

The study population included all consenting adult patients (>18 years) attending these 13 PC clinics in Nairobi County. The family medicine department at the hospital was excluded. Children and those who were too sick or unable to participate were also excluded from the study. Every month, approximately 15 300 patients were seen across all the clinics. The sample size calculation was, therefore, based on a population of 20 000 patients, as sample size calculations do not change markedly in populations over this size. Patient satisfaction was assumed to be 70%,^10,29,30^ confidence intervals 95% and margin of error 5%. Using these assumptions in Fischer’s formula for one proportion, the minimum sample size was 318 patients. The final sample size required was 350 after an adjustment of 10% for incomplete responses.

### Sampling strategy

The number of patients selected per clinic was proportional to the clinic’s workload, as measured by the monthly headcount by using the daily register as a master frame. Consenting participants were randomly selected by using computer-generated random numbers until the required sample was obtained. It took a period of 2 months to collect the data from all 13 PC clinics, which were spread all over Nairobi.

### Data collection tool

The GPAQ-R2 tool is a validated tool that is used worldwide for quality assessment of PC service delivery.^31,32^ The GPAQ-R2 tool consists of 46 multiple choice and Likert-scale questions (Appendix 1). The Likert scales are all scored differently depending on the type of questions asked. To adapt this already validated tool to the local context, three family medicine experts validated the content. They were asked to give feedback on whether the questions were relevant to the local context and phrased appropriately. The questionnaire was then piloted in a similar PC clinic, which was not included in the study, with a group of 35 patients to assess its face validity, acceptability and feasibility. No changes were made to the GPAQ-R2 questionnaire as a result of the validation and piloting.

### Data collection process

Data was collected by trained research assistants in the PC clinics who provided the consenting patients with the self-administered questionnaire after their consultation. All the requested participants completed the survey in English. A recent study carried out at the same PC clinics revealed that the majority of patients were English speaking, and consultations were also conducted in English.^33^ The research assistant was available to provide help and clarification in Swahili if needed.

### Data analysis

The researchers aligned the GPAQ-R2 questions with key domains of PC service delivery as shown in Table 1.

**TABLE 1:** Relationship of General Practice Assessment Questionnaire questions to key domains of service delivery.

Domains	Number of items	GPAQ questions
Socio-demographics	5	42–46
Access to the practice	10	12–19, 22–23
Consultation with the GP	8	1–8
Confidence in the patient – GP relationship	2	9–10
Care enablement	3	37–39
Care continuity	4	20, 21, 28, 29
Overall satisfaction with the GP and practice	3	11, 40, 41

GP, general practitioner; GPAQ, General Practice Assessment Questionnaire.

The literature on GPAQ-R2 does not calculate composite scores for different domains or constructs. The questions therefore are reported and interpreted individually in the results, but grouped together into the domains described in Table 1.

**TABLE 2:** Socio-demographic characteristics and health status of the patients (*N* = 378).

Variables	Total	
	*n*	%
**Gender**		
Male	146	38.6
Female	232	61.4
**Age in years**		
18–44	289	76.4
45–64	82	21.7
65 and over	7	1.9
**Employment status**		
Employed	280	74.1
Unemployed	20	5.3
Studying	28	7.4
Others	50	13.2
**Long-standing health condition**		
Yes	69	18.3
No	275	72.7
Don’t know/can’t say	34	9.0

Data was entered into an Excel spreadsheet and analysed by using the Statistical Package for Social Sciences (SPSS version 25). All data were categorical, and therefore descriptive analysis was reported as frequencies and percentages. Three variables that measured overall satisfaction with the quality of service delivery were compared with the demographic variables by using Pearson’s Chi Square test. These variables were: ‘Would you be completely happy to see this GP again?’ ‘Overall, how would you describe your experience of your GP surgery?’ and ‘How likely are you to recommend your GP surgery to friends and family if they need similar care or treatment?’

### Ethical consideration

The study was approved by the Research and Ethics Committee (REC) of the Aga Khan University Hospital, Nairobi (reference number: 2018/REC-137[v2]), and complied with the ethical guidelines.

## Results

Table 2 shows the socio-demographic characteristics of the 378 respondents. In the category on employment status, the item ‘others’ refers to respondents who stayed at home because they were retired, homemakers or chronically ill.

The majority of participants were under 45 years of age (289, 76.4%), women (232, 61.4%), employed (280, 74.1%) and without chronic diseases (275, 72.7%).

The majority (367, 97.1%) would be happy to see the GP again and were satisfied (373, 98.6%) with their overall experience of the practice. They were also very likely to recommend the practice to friends or family (311, 83.0%).

Table 3 shows high levels of satisfaction with the consultation, confidence in the provider–patient relationship and care enablement. High level of confidence was expressed (283, 74.9%) with the GPs ‘honesty and trustworthiness’. On the other hand, 58 (15.3%) patients showed some doubt about the GPs’ ability to maintain confidentiality. High proportions of patients felt the GP enabled them to understand (289, 76.5%) and cope with their health problems (288, 76.2%) and guided them in lifestyle changes (288, 76.2%).

**TABLE 3a:** Patients’ perspective on the consultation, relationship with the general practitioner and care enablement (*N* = 378).

Consultation with the GP	Very good		Satisfactory		Poor		Does not apply	
	*n*	%	*n*	%	*n*	%	*n*	%
Putting you at ease	325	86.0	50	13.2	2	0.5	1	0.3
Being polite and considerate	343	90.7	35	9.3	0	0.0	0	0.0
Listening to you	339	89.6	38	10.1	1	0.3	0	0.0
Giving you enough time	338	89.5	38	10.1	1	0.2	1	0.2
Assessing your medical condition	338	89.5	33	9.1	4	1.3	3	0.8
Explaining your condition and treatment	327	86.5	43	11.4	4	1.1	3	1.0
Involving you in decisions about your care	322	85.2	44	11.6	6	1.6	6	1.6
Providing or arranging treatment for you	331	88.0	40	10.5	2	0.5	5	1.0

GP, general practitioner

**TABLE 3b:** Patients’ perspective on the consultation, relationship with the general practitioner and care enablement (*N* = 378).

Confidence in the patient–GP relationship	Definitely		To some extent		None		Don’t know/can’t say	
	*n*	%	*n*	%	*n*	%	*n*	%
Confidence in GPs’ honesty and trustworthiness	283	74.9	79	20.9	4	1.0	12	3.2
Confidence in GPs’ commitment to confidentiality	295	78.0	58	15.3	1	0.3	24	6.4

GP, general practitioner

**TABLE 3c:** Patients’ perspective on the consultation, relationship with the general practitioner and care enablement (*N* = 378).

Care enablement – how well the GP enabled the patient to:	Very well		Unsure		Not very well		Does not apply	
	*n*	%	*n*	%	*n*	%	*n*	%
Understand your health problems	289	76.5	53	14.0	14	3.7	22	5.8
Cope with your health problems	288	76.2	51	13.5	11	2.9	28	7.4
Keep yourself healthy	288	76.2	47	12.4	13	3.4	30	8.0

GP, general practitioner

**FIGURE 1:**
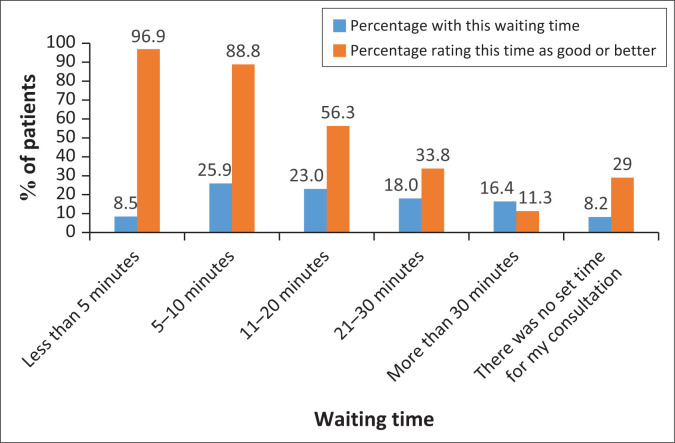
Waiting time and patient satisfaction (*N* = 378).

Table 4 presents the results for access and support of continuity of care. The majority (366, 96.8%) found the receptionist helpful and the clinic opening hours convenient (276, 73%). There was no clear preference expressed for additional or alternative opening hours. Overall, 294 (77.8%) patients were satisfied with the waiting time, 85% of patients waited less than 30 min and 25% less than 10 min (Figure 1).

**TABLE 4:** Access to the practice and general practitioner, and continuity of care (*N* = 378).

Variables	Total	
	*n*	%
**Access to the practice and GP**		
**How easy is it to get through to someone at your GP practice on the phone?**		
Easy	187	49.5
Not easy	28	7.4
Haven’t tried	163	43.1
**How easy is it to speak to your doctor or nurse on the phone at your GP practice?**		
Easy	143	37.8
Not easy	28	7.4
Haven’t tried	207	54.8
**How do you normally book your appointments at your practice?**		
In person	214	56.6
By phone	98	25.9
Online	14	3.7
Doesn’t apply	109	28.8
**Which of the following methods would you prefer to use to book appointments at your practice?**		
In person	180	47.6
By phone	193	51.1
Online	85	22.5
Doesn’t apply	57	15.1
**Willing to see any doctor: How quickly do you usually get seen?**		
Same day or next day	229	60.6
2–4 days	21	5.6
5 days or more	5	1.3
I don’t usually need to be seen quickly	35	9.3
Don’t know, never tried	88	23.3
**How do you rate how quickly you were seen?**		
Excellent	166	43.9
Good	62	16.4
Satisfactory	37	9.8
Poor	13	3.5
**Continuity of care**		
**Is there a particular GP you usually prefer to see or speak to?**		
Yes	98	25.9
No	274	72.5
There is usually one doctor in my surgery	6	1.6
**Want to see a particular doctor: How quickly do you usually get seen?**		
Same or next day	165	43.7
2–4 days	23	6.1
5 days or more	10	2.6
I don’t usually need to be seen quickly	41	10.8
Don’t know, never tried	139	36.8
**How do you rate how quickly you were seen?**		
Excellent	165	43.5
Good	59	15.7
Satisfactory	48	12.7
Poor	15	4.0
Does not apply	91	24.1

GP, general practitioner.

Of all the participants, 317 (83.9%) expressed the importance of making an advanced booking for their appointment, but only 183 (48.4%) felt that this was easy to do, and 149 (39.4%) had not tried to do so. Almost half of the participants (186, 49.2%) were of the view that in case of an emergency, they would be able to see the GP on the same day. The majority of patients (274, 72.5%) did not express the need to see or speak to a particular GP.

Table 5 shows the relationship between measures of overall satisfaction and the patient socio-demographics. There was no association between patient socio-demographics and their overall experience of the practice. However, there was an association between their employment status and being happy to see the same GP again, as well as willingness to recommend the practice to friends and family. Post hoc analysis showed that those in employment were significantly more satisfied than those that were unemployed, studying, retired or home for other reasons. There was no association with any of the other variables such as age, gender or presence of a chronic condition.

**TABLE 5:** Relationship between socio-demographics and overall patient satisfaction with quality of service delivery.

Variable	Would you be completely happy to see this GP again?			How likely are you to recommend your GP practice to someone else?						
	Yes		Chi-square/*p*-value	Likely		Unlikely		Don’t know		Chi-square/*p*-value
	*n*	%		*n*	%	*n*	%	*n*	%	
**Gender**										
Male (*N* = 146)	142	97.3	*χ*^2^ = 0.024	125	85.6	3	2.1	18	12.3	*χ*^2^ = 1.966
Female (*N* = 232)	225	97.0	*p* = 0.876	186	80.2	5	2.2	41	17.7	*p* = 0.374
**Age in years**										
18 to 44 (*N* = 289)	281	97.2	*χ*^2^ = 4.134	240	83.0	6	2.1	43	14.9	*χ*^2^ = 12.140
45 to 64 (*N* = 82)	80	97.6	*p* = 0.247	67	81.7	1	1.2	14	17.1	*p* = 0.059
65 and over (*N* = 7)	5	71.4	-	4	66.7	1	16.7	1	16.7	-
**Employment status**										
Employed (*N* = 280)	275	98.2	-	241	86.1	2	0.7	37	13.2	-
Unemployed (*N* = 20)	19	95.0	*χ*^2^ = 39.801	15	75.0	0	0.0	5	25	*χ*^2^ = 71.212
Studying (*N* = 28)	26	92.9	*p* < 0.001	20	71.4	3	10.7	5	17.9	*p* < 0.001
Others (*N* = 50)	47	94.0	-	35	70.0	2	4.0	12	24.0	-
**Long-standing health condition**										
Yes (*N* = 69)	65	94.2	*χ*^2^ = 2.552	55	79.7	3	4.3	11	15.9	*χ*^2^ = 3.189
No (*N* = 275)	269	97.8	*p* = 0.279	230	83.6	4	1.5	41	14.9	*p* = 0.527
Don’t know (*N* = 34)	33	97.1	-	26	76.5	1	2.9	7	20.6	-

GP, general practitioner.

## Discussion

The quality of service delivery in these private sector PC clinics in Nairobi, was high as measured from the patients’ perspective. Patients were particularly satisfied with their consultations, care enablement, confidentiality and their overall experience of the practice. Lower levels of satisfaction were expressed in terms of overall access to the practice, access to a particular GP and for emergencies. Patients did not express a strong desire for relational continuity and thought it was easier to see any GP rather than a specific GP. The practice population mostly consisted of young and middle-aged patients, who were employed and without chronic conditions. Patients who were employed were more satisfied, but age, gender and having a chronic condition had no association with overall satisfaction.

The questions on the consultation covered key aspects of person-centredness such as listening, providing enough time to tell your story, explaining the problem, involvement in decision-making and enabling self-care.^18^ This high satisfaction with the consultation therefore also appeared to reflect an experience of person-centredness. Other studies carried out in Canada, United Kingdom, Bangladesh and Nigeria realised high satisfaction with the consultation.^13,21,34,35^ Despite this implication, other studies in the region have suggested that patients can be very satisfied with consultations that lack person-centredness.^36,37^ Therefore, it may be important to verify this finding by assessing actual recordings of the consultation against more objective criteria.^36^ Patients attending private practice may assume that care is of high quality and feel more satisfied, even if these assumptions are not objectively verified. In this private PC settings, being able to consult a doctor may also have been sufficient to satisfy the patients, as in the public sector they would see a nurse or clinical officer (mid-level doctor).

In this study, patients were very satisfied with the services provided, and the skewing of the practice population towards healthy younger adults suggests that patients selectively used the clinics for minor episodic acute ailments. A previous study in the same clinics showed that patients had limited expectations of these GPs in terms of the comprehensiveness of services available.^33^ For example, patients had low confidence in the GPs’ ability to manage tuberculosis, human immunodeficiency virus (HIV), cancer, elderly patients, mental disorders, antenatal and reproductive health care.^38^

High levels of confidence were expressed in the doctor–patient relationship, as shown by the GPs’ integrity and the ability to maintain confidentiality. The confidence and trust placed by patients in these private GPs was much higher than that reported by patients in the public sector, where care may be more doctor-centred as well as lacking in privacy, confidentiality and resources.^23^

Continuity of care is thought to be a hallmark of quality PC^7^ and yet the majority of patients in this study did not express a preference to see a particular doctor. The lack of desire for continuity with a specific GP may imply that whilst patients had easy access to the services, they did not regard the GP as their sole or preferred PC provider. It may be that older patients, with a need for chronic care, would value relational continuity more, but this group was a minority in the practice population. The lack of commitment to a specific relationship may also be because of the lack of compulsory gatekeeping in this private health system and the insurance coverage that enabled the ability to seek help directly from the family physicians or specialists at the tertiary hospital. In the broader Kenyan context, continuity of care may not be seen as a key goal of service delivery in the health system. Therefore, patients may not expect or value continuity so much. In the United Kingdom, patients have an expectation of relational continuity with their GP, maybe because they register with them specifically and complain of not being able to see their own GP easily.^21^

Their expectations in terms of telephonic consultation and appointment systems also appeared to be lower than in high-income settings.^39^ These clinics are all walk-in clinics and although patients do have the opportunity to call and make a booking in advance, this approach was not necessarily an advantage, as around half of the patients had never tried to phone the practice, book ahead or speak to the GP on the phone. Although patients expressed an interest in booking by phone, few had actually attempted to do so. One of the reasons for this appeared to be the convenient opening hours and the availability of the GP. Telephonic consultations, which are becoming popular in high-income countries,^40^ were not yet part of service delivery in this context. This could also be because of the fact that insurance in Kenya does not reimburse for tele-health.

In these PC clinics, almost half of the participants expressed doubt that they would be able to see the GP on the same day in case of an emergency. On the other hand, it was also noted that half of the participants had not tried to reach the GP as a matter of urgency. This could be explained by the fact that most patients had private medical insurance, which allowed them to seek care from any emergency department as well as the perception that GPs do not manage emergencies.^33^

Most of these PC clinics operated during the day, evening and weekends. Therefore, it was not surprising that the majority felt that the opening times were convenient and waiting times acceptable. Access and utilisation of services in these clinics were favourable for the employed, who were more satisfied and made up the majority of patients. Other studies in PC in the region have found lower levels of satisfaction with access, and this may be because they were in the public sector; where opening times may not be convenient, appointment systems may be dysfunctional and waiting times are much longer.^41,42,43^

Employed patients had a higher level of satisfaction in this study. Although there is some evidence that higher levels of patient satisfaction are seen in those coming from higher socio-economic backgrounds,^24,44^ this finding needs to be further explored to understand why unemployed and other patients were significantly less satisfied.

Although the lack of correlation between having a chronic condition and overall satisfaction was also found in private practice in South Africa,^45^ the small numbers of patients with chronic conditions reduced the power to test this relationship. The assumption that, older patients with chronic diseases and multi-morbidity, were most likely attending the tertiary hospital has also been noted in a tertiary care hospital in Australia.^46^ This again reflects the limited comprehensiveness of these PC clinics.^33^ In effective health systems, the management of chronic diseases is an essential feature of PC because of the high volume of patients, easy access and need for continuity. Health systems are more cost-effective when chronic conditions are managed in PC.^1^ The routine management of patients with chronic conditions in a tertiary hospital setting represents a missed opportunity for effective PC.^1,46^

Interestingly, the number of elderly patients (>65 years) in this study was very small, and this may reflect the life expectancy in Kenya of 67 years or the lack of health insurance when retired.^47^ Perhaps the perception that GPs were less capable of managing the elderly could have also contributed to the low numbers as was shown in the previous study carried out at the same settings.^33^ It is also possible that elderly patients were being referred to the specialists at the tertiary care hospital for chronic conditions or had retired to their homes in the rural areas, which is a common practice in Kenya.^33^ However, in this study with a more affluent, educated population and with good access to healthcare, one might expect patients to live longer than the Kenyan average.

### Limitations

The General Practice Assessment Questionnaire (GPAQ) was a validated tool, which was adapted to the African context, and most of the questions were applicable to the study context. The question on ethnicity that was constructed within the context of the United Kingdom created some confusion, and hence it was removed from the analysis. Collecting the data in the facility might have put some pressure on the participants to give a more favourable response. To mitigate this, data was collected by a neutral research assistant who was not known to the participant or associated with the facility.

The findings of this study may be generalised to other PC clinics associated with this organisation in East Africa. It cannot be generalised to the public sector and may be limited in the wider private sector, as organisations differ in the way services are organised and offered.

### Recommendations

Because of the complex relationship of the patient’s perspective to quality of service delivery, it would be useful to assess service delivery using additional methods, such as the PC assessment tool,^42^ to provide a more in-depth evaluation.^7^ Ultimately, this private sector health system may need to consider whether, despite high levels of satisfaction, the PC clinics are a resource that can be developed further by incorporating the services of the family physicians who are more trained in providing comprehensive care.^48^

## Conclusion

Patients were highly satisfied with the service delivery at these private sector PC clinics in Nairobi, Kenya. Services were easily accessible, although there was little expectation of relational continuity. Patients were satisfied with the GPs’ consultation, care enablement and the GP–patient relationship. However, the practice population was skewed towards younger and healthier adults, and it appeared that services were not comprehensive. High levels of satisfaction may mask inadequacies in terms of care for people with emergencies, chronic conditions and multi-morbidity. Further studies are needed to evaluate whether these private sector PC clinics provide high-quality, cost-effective and comprehensive services.
